# Electroconvulsive therapy in a patient with an implanted sacral neurostimulator: a case report on safe administration and short-term outcomes

**DOI:** 10.3389/fpsyt.2025.1563519

**Published:** 2025-08-15

**Authors:** Jeet Janak Patel, Marcela Carbajal-Tamez, William Baumgartner, Cristina Abraham, Edison Leung, João Quevedo

**Affiliations:** ^1^ Center for Interventional Psychiatry, Faillace Department of Psychiatry and Behavioral Sciences at McGovern Medical School, University of Texas Health Science Center at Houston, Houston, TX, United States; ^2^ Anesthesiology, Critical Care and Pain Medicine, McGovern Medical School, The University of Texas Health Science Center at Houston, Houston, TX, United States

**Keywords:** electroconvulsive therapy (ECT), overactive bladder (OAB), implanted medical devices (IMD), safety, case report, depression, adult, treatment outcomes

## Abstract

We present a case of a 35-year-old patient with treatment-resistant depression and an implanted sacral neurostimulator for overactive bladder (OAB). The patient experienced an exacerbation of depression with suicidal ideation and failed multiple medication trials. Due to her significant history of adverse medication reactions and the severity of her condition, electroconvulsive therapy (ECT) was selected as a treatment option despite concerns about the safety of administering ECT with a sacral neurostimulator. To minimize potential risks, the device was placed in Magnetic Resonance Imaging (MRI) mode during each ECT session, successfully avoiding electrical interference. She underwent three ECT sessions, which resulted in significant improvement in depressive symptoms and resolution of suicidal ideation without adverse effects on the device’s integrity or OAB symptoms. This case highlights the feasibility and safety of ECT in patients with implanted sacral neurostimulators, emphasizing the importance of precautionary measures and individualized patient assessment. Further research is needed to explore the long-term effects of ECT on such devices and their impact on OAB.

## Introduction

Electroconvulsive therapy (ECT) is a well-established treatment for severe and treatment-resistant depression (TRD), with open-label remission rates around 48% for non-psychotic cases, significantly surpassing those achieved through pharmacologic augmentation strategies (1). Despite its efficacy, ECT is often withheld in patients with implanted electronic medical devices due to theoretical concerns about induced currents, tissue heating, or device malfunction. One such device is the sacral neuromodulator system InterStim X^®^, increasingly used to manage refractory overactive bladder (OAB) and urinary urge incontinence when behavioral modifications and pharmacotherapy have failed (2, 3). With over 300,000 implants globally (4) and device longevity frequently exceeding a decade, evaluating the safety implications of administering ECT to patients with implanted sacral neurostimulators is of growing clinical relevance (5).

Although electromagnetic modeling suggests potential risks from ECT currents coupling with distant leads, empirical evidence from other neurostimulators, such as deep-brain, spinal cord, and cardiac stimulators, suggests that the risk is negligible when stimulation is suspended and leads are anatomically distant (6–11). However, only one prior case has specifically examined ECT in a patient with a sacral neuromodulator (11), leaving clinicians with limited guidance. This case report addresses that gap by describing the successful administration of bitemporal ECT in a woman with chronic InterStim X^®^ therapy, treated for TRD and acute suicidality. We emphasize the practical peri-procedural precautions that supported safe treatment delivery, underscoring the feasibility of ECT in patients with implanted sacral neurostimulation systems.

## Case presentation

The patient is a female in her mid-30s with a long-standing history of recurrent major depressive disorder (MDD), generalized anxiety disorder (GAD), ADHD, and PTSD. At the time of presentation, she had been admitted due to increased symptom severity and active suicidal ideation. Her medical history includes a traumatic brain injury requiring ICU admission and resulting in a coma; obesity managed with prior bariatric surgery; recurrent cystitis; and overactive bladder (OAB) previously treated with botulinum toxin and currently managed with an implanted neuromodulation device. She lives independently and serves as the primary caretaker of a minor. No relevant family psychiatric history was reported.

**Figure 1:**
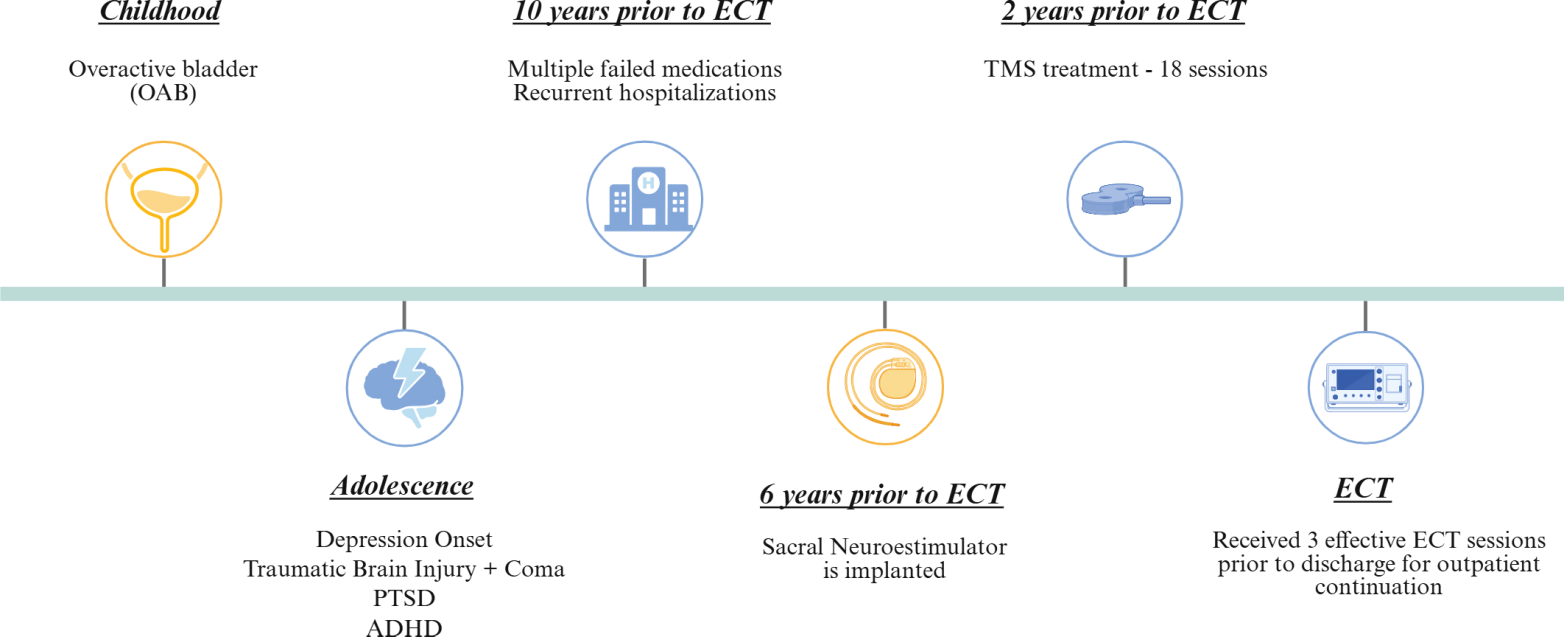
Clinical timeline summarizing the patient's psychiatric and neuromodulation history. The patient’s clinical course is shown chronologically, beginning in childhood with overactive bladder (OAB), followed by psychiatric symptom onset and comorbidities in adolescence, including depression, traumatic brain injury with coma, PTSD, and ADHD. Over the subsequent decade, the patient experienced multiple medication failures and hospitalizations. A sacral neurostimulator was implanted 6 years prior to electroconvulsive therapy (ECT), and transcranial magnetic stimulation (TMS) was trialed 2 years prior to ECT. The patient ultimately received a successful course of ECT with three effective inpatient sessions prior to discharge for outpatient continuation. Created in BioRender. Carbajal Tamez, M. (2025) https://BioRender.com/2umw1qi.

She was first diagnosed with MDD in her adolescence following a suicide attempt and has since received psychiatric care for over a decade, including individual therapy, psychotropic medications, and neuromodulation therapies. She reports approximately 20 prior psychiatric hospitalizations due to symptom exacerbation, and has a previous history of two suicide attempts. Over the years, she has tried over 25 psychotropic agents across multiple classes. Many were discontinued due to side effects (e.g., TCAs, SSRIs, mood stabilizers, atypical antipsychotics), while others failed to achieve sustained symptom relief despite adequate dosing and duration according to the American Psychiatric Association Guidelines (12). She also attempted transcranial magnetic stimulation (TMS) therapy at an external facility but discontinued after 24 sessions due to a perceived lack of benefit. Detailed protocol parameters and outcome measures were unavailable at the time of evaluation. A summary of her clinical timeline can be found in **Figure 1**.

During her initial evaluation at our facility, the clinical team reviewed her extensive medication history, prior augmentation strategies, and limited access to detailed external records. Given her history, past suicidal attempts, and current severity, she was considered a candidate for ECT and admitted for inpatient care.

On physical examination, she was alert and oriented, with normal vital signs and no focal neurological deficits. Mental status examination revealed depressed mood, passive suicidal ideation, intact insight and judgment, and no evidence of psychosis or cognitive impairment. Her admission medication regimen included lurasidone, haloperidol, gabapentin, alprazolam, and eszopiclone. The patient refused changes to her medications during hospitalization, citing prior poor tolerability and perceived ineffectiveness; the treatment team respected her autonomy and maintained the regimen. To preserve patient anonymity, treatment details are summarized in **Table 1** by drug class without specific dosages or dates, along with a response summary.

**Table 1:** Summary of prior pharmacologic and interventional treatments.

Strategy	Examples	Reason for discontinuation	Response summary
SSRIs, SNRIs	Citalopram, Sertraline, Venlafaxine	Anxiety worsening, side effects	Ineffective/poorly tolerated
TCAs	Amitriptyline, Doxepin	Side effects	Early discontinuation
Atypical Antipsychotics	Quetiapine, Olanzapine, Paliperidone	Side effects or partial response	Partial benefit (Ongoing)
Mood stabilizers	Valproate, Lamotrigine, Lithium	Inadequate response	Augmentation trials, partial benefit
Anxiolytics	Clonazepam, Lorazepam, Alprazolam	Partial relief	Partial benefit (Ongoing)
Hypnotics	Zolpidem, Eszopiclone	Varied tolerability	Limited utility
Interventional Strategies	TMS	18 sessions	Ineffective

During hospitalization, diagnostic evaluation included repeat physical and psychiatric examinations, confirming passive suicidal ideation without psychosis, routine laboratory testing, and medical clearance for ECT. Psychiatric symptom severity was regularly monitored using the Patient-Health Questionnaire (PHQ-9) alongside daily psychiatric evaluations and nursing assessments. The patient also actively participated in milieu therapy, psychoeducational sessions, and substance abuse counseling. A diagnostic challenge was the limited availability of detailed prior treatment records, necessitating reliance on patient report and clinical judgment for treatment planning.

**Figure 2:**
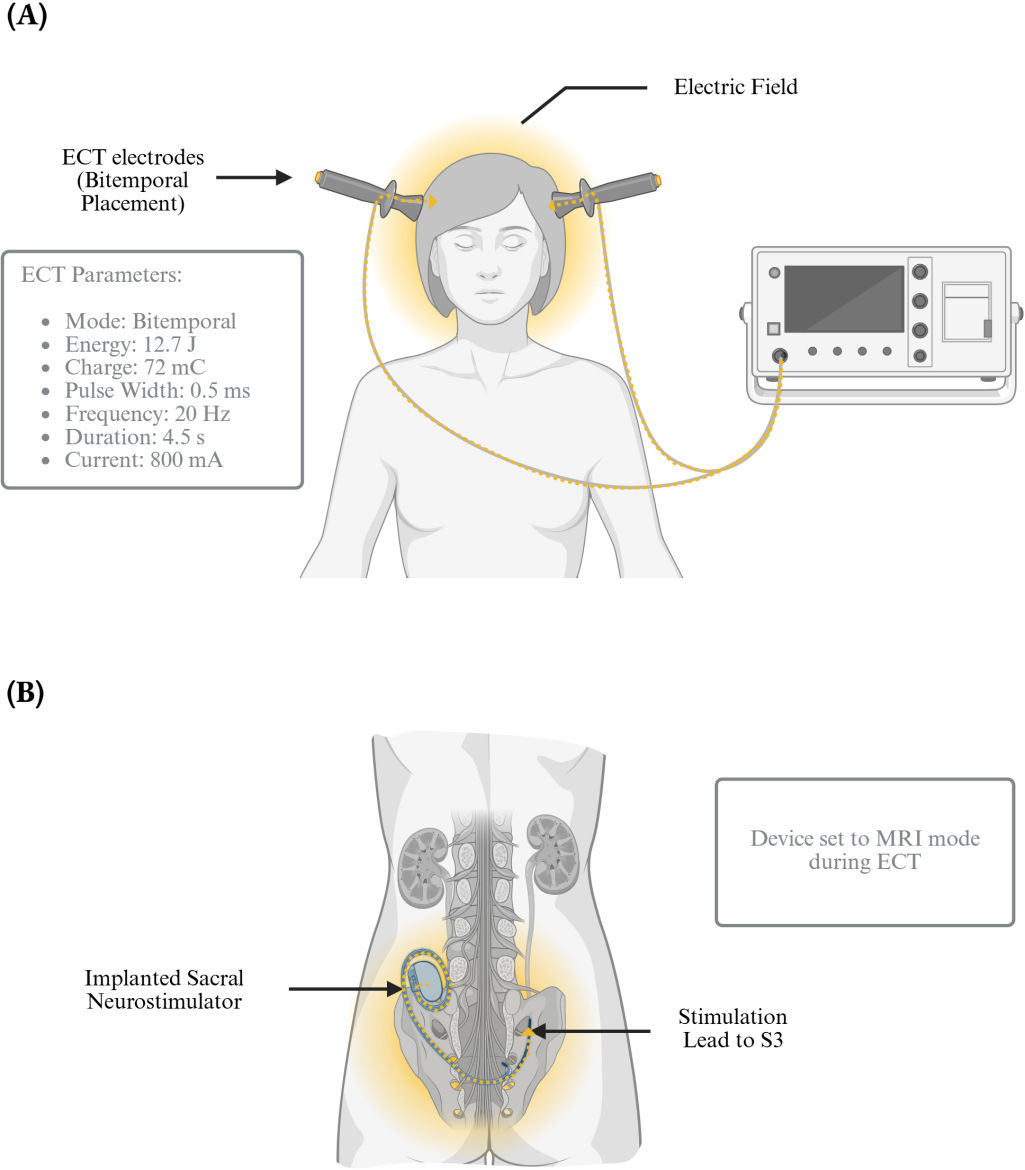
Electroconvulsive therapy administration in a patient with an implanted sacral neurostimulator. **(A)** Frontal view of bitemporal ECT administration, showing electrode placement, electric field distribution, and stimulation parameters including mode, energy, charge, pulse width, frequency, duration, and current. **(B)** Posterior view of the patient depicting an implanted sacral neurostimulator with a lead targeting the S3 nerve root. During ECT, the device was placed in MRI mode to minimize potential interference or adverse effects. Created with BioRender.com. Carbajal Tamez, M. (2025). https://BioRender.com/20tyitk.

A key consideration in procedure planning was her implanted sacral neurostimulation device (InterStim X, Medtronic), placed six years prior for OAB management. According to product documentation (13), the safety of ECT in patients with this device is unestablished due to the theoretical risks of induced electrical currents causing device malfunction or tissue damage. However, given the anatomical distance between the ECT electrodes and the implanted device (**Figure 2**), the risk was assessed as minimal. To further mitigate potential risks, the device was set to Magnetic Resonance Imaging (MRI) mode—suspending stimulation—before each ECT session and reactivated afterward to restore programmed settings. The plan was reviewed with the patient, who consented to proceed with a low-dose ECT regimen incorporating these precautions.

The patient underwent three ECT sessions every other day using a Mecta Σigma ECT machine. Anesthesia was induced with propofol, and muscle relaxation was achieved with succinylcholine. Bitemporal electrode placement was selected. Seizure threshold was established during the first session using the following parameters: pulse width of 0.5 ms, frequency 20 Hz, duration 4.5 seconds, current 800 mA, energy 12.7 Joules, and charge 72 mC. The second session charge was increased to 1.5 times the initial seizure threshold, reaching 96 mC (energy of 16.9 Joules while keeping all other parameters constant. This same configuration was maintained for the third session. **Figure 2** summarizes the ECT protocol employed.

The patient experienced a significant improvement following the first ECT session, with her suicidal ideation resolving completely. Her pre-treatment Patient Health Questionnaire - 9 score of 4 decreased to 2 by the third session. At discharge, she reported substantial symptom relief, denied any ongoing suicidal thoughts, and was stable on daily psychiatric and nursing evaluations. The patient was subsequently discharged to continue ECT as an outpatient at a frequency of three sessions per week.

No adverse effects were observed from ECT at the time of discharge. The patient’s OAB symptom diary and urinary frequency chart showed no changes from baseline. One month post-intervention, and during her outpatient treatment, the patient attended a previously scheduled surgical device revision with her urologist and a Medtronic representative, who confirmed device integrity, remaining battery life (6–12 months), and unchanged stimulation parameters.

## Discussion

To our knowledge, empirical reports describing the administration of electroconvulsive therapy (ECT) in patients with an implanted sacral neuromodulation device (such as the Medtronic InterStim system) remain rare in the published literature. This case report highlights the safe and effective administration of electroconvulsive therapy (ECT) in a patient with an implanted sacral neurostimulator. Our findings align with previous research (6–11), including the case reported by Hiremani RM (11), demonstrating that ECT can be safely administered in patients with implanted devices such as sacral neurostimulators, deep brain stimulators, cardiac pacemakers, and spinal cord stimulators. These devices, despite their complex functionalities, do not necessarily contraindicate ECT and might be able to withstand the electrical demands of this procedure, provided that appropriate precautions are taken.

The risk of lead heating during ECT is proportional to pulse width, current density, and tissue conductivity. Prior bench testing of DBS hardware exposed to ECT-like pulses produced < 1 °C temperature change at worst-case parameters. Sacral leads are shorter, lie in high-resistance soft tissue, and—when the generator is disabled—form an open circuit, further minimizing current flow. MRI-mode on the InterStim X^®^ additionally deactivates telemetry, preventing spurious resets.

In this case, precautions were taken by putting the patient’s InterStim device in MRI mode before each ECT session, which turned off the stimulation and minimized the risk of potential electrical interference. This strategy, coupled with careful patient monitoring, allowed us to successfully conduct three sessions without any adverse outcomes related to the stimulator. Notably, our case contributes to the limited literature on this topic by providing additional evidence supporting the compatibility of ECT with sacral neurostimulation devices. Aligning with previous research documented in > 30 DBS recipients (6), cardiac pacemakers (7), cochlear implants (8), and spinal stimulators (9). Across these series, no permanent hardware damage or stimulation-related injury has been recorded, supporting a class-effect of low risk when stimulation is suspended.

While our report demonstrates the short-term safety of ECT in a patient with an implanted sacral neurostimulator, it is essential to acknowledge that the primary limitation of this report includes its single-case nature and the observation of exclusively short-term safety. Long-term effects on device integrity remain unexplored. Additionally, the effect of ECT on OAB symptoms has not been comprehensively studied, and objective urodynamic data were unavailable.

## Future directions

Prospective registries are needed to quantify long-term device longevity, lead integrity, and bladder outcomes after repeated ECT exposures. Integration of impedance logging into routine post-ECT follow-up could provide objective safety metrics. In the interim, shared decision-making that balances psychiatric urgency against theoretical device risks remains essential.

## Patient perspective

The patient shared that receiving ECT was initially a source of anxiety, particularly due to the presence of her implanted sacral neurostimulator. However, she reported significant relief after treatment, expressing appreciation for the careful planning and collaborative communication from the care team regarding her device safety, and felt reassured throughout the process.

## Conclusion

In conclusion, this case reinforces existing evidence that with thoughtful multidisciplinary planning, ECT can be safely administered to patients with implanted devices, including sacral neurostimulators. It also emphasizes the importance of individualized patient assessment and meticulous precautionary measures when implementing ECT in such cases. Further research is essential to expand our understanding of both the long-term device safety and the broader implications of ECT on coexisting conditions like OAB.

## Learning points

- Safe Administration of ECT in Patients with Implanted Devices: With appropriate precautions, such as placing the device in MRI mode, ECT can be safely administered in patients with implanted sacral neurostimulators without compromising device integrity or functionality.
- Importance of Individualized Patient Assessment: Comprehensive assessment and tailored treatment plans are critical when managing patients with complex medical histories and implanted devices to minimize risks and optimize outcomes.
- Short-term Safety Demonstrated; Long-term Effects Unknown: While short-term outcomes were positive in this case, further research is needed to explore the long-term impact of ECT on implanted neurostimulators and associated conditions like OAB.
- Potential Neural Modulation Implications: ECT may potentially influence symptoms related to coexisting conditions such as OAB, warranting additional study into its broader therapeutic effects beyond primary psychiatric indications.

## Data Availability

The original contributions presented in the study are included in the article/**Supplementary Material**. Further inquiries can be directed to the corresponding author.
